# Reconstituted and ensiled corn or sorghum grain: Impacts on dietary nitrogen fractions, intake, and digestion sites in young Nellore bulls

**DOI:** 10.1371/journal.pone.0237381

**Published:** 2020-08-07

**Authors:** Breno de Castro Silva, Marcos Vinicius Carneiro Pacheco, Letícia Artuzo Godoi, Herlon Menegueli Alhadas, Jéssica Marcela Vieira Pereira, Luciana Navajas Rennó, Edenio Detmann, Pedro Veiga Rodrigues Paulino, Jon Patrick Schoonmaker, Sebastião de Campos Valadares Filho

**Affiliations:** 1 Department of Animal Science, Universidade Federal de Viçosa, Viçosa, Minas Gerais, Brazil; 2 Cargill Animal Nutrition / Nutron, Campinas, São Paulo, Brazil; 3 Department of Animal Sciences, Creighton Hall of Animal Sciences, Purdue University, West Lafayette, Indiana, United States of America; Murdoch University, AUSTRALIA

## Abstract

Two experiments were conducted: (1) to evaluate the effect of ensiling time and grain source on dietary nitrogen fractions; and (2) to verify the influence of concentrate level, processing method and grain source on intake, microbial efficiency, and digestibility by young Nellore bulls. In Experiment 1, corn and sorghum grains were milled, reconstituted to 35% moisture, and ensiled in a bag silo for 10 different times. There were three replications per ensiling time and grain source. Samples from each replication were analyzed in triplicate for total nitrogen (N), non-protein nitrogen (NPN), soluble N, insoluble N, and neutral detergent insoluble nitrogen (NDIN). In Experiment 2, five Nellore bulls were used in a 5 × 5 Latin square design. Four diets were comprised of 28.4% corn silage, 10.7% supplement, and 60.9% dry ground corn, dry ground sorghum, reconstituted and ensiled corn, or reconstituted and ensiled ground sorghum. An additional diet comprised of 45% corn silage, 10.7% supplement, and 44.3% dry ground corn (Roughage+) was used. Each experimental period lasted 22 days, with an adaptation period of 14 days followed by 5 days of total feces and urine collection and 3 days of collecting omasal samples. Data were analyzed using the MIXED procedure of SAS 9.4. The reconstitution and ensiling process reduced (*P* < 0.05) the insoluble N fraction, increased (*P* < 0.05) non-protein nitrogen of corn and sorghum grains, tended (*P* = 0.052) to increase microbial efficiency, and increased (*P* < 0.05) intestinal and total digestion of dry matter (DM), organic matter (OM), crude protein (CP), and starch. The concentrate level affected neither (*P* > 0.05) DM intake nor rumen pH. On the other hand, bulls fed diets based on 72% concentrate showed greater (*P* < 0.05) DM, OM, and CP digestibility compared with those fed a diet based on 55% concentrate. In addition, animals fed diets based on corn grains (both reconstituted and ensiled or dry) presented greater (*P* < 0.05) intestinal and total starch digestion compared to those fed sorghum grain. Therefore, the reconstitution process can reduce the insoluble N fraction and increase nutrient availability.

## Introduction

Nowadays, cereal grains represent more than 51% of feedlot diets composition [1, 2], with corn and sorghum being the most used grains in those diets [3]. However, grain price volatility and metabolic disorders accompany the use of high grain levels in beef cattle diets. These issues can result in losses to the production system [4, 5]. For these reasons, some medium and small farms in Brazil tend to adopt lower levels of concentrate in their feedlot diets [6]. Furthermore, the digestibility and feed value of cereal grains are dependent on how their starch structure interacts with prolamin, a water insoluble protein that is resistant to enzymatic digestion. Prolamins form a protein body that can surround starch granules, making starch less digestible [7, 8]. Even when partially broken or milled, the prolamin concentration in the grain—in its native, dry form—may show some resistance to microbial or intestinal degradation and prevent the use of high-grain diets from improving performance [7, 9].

Thus, more elaborate grain processing methods, such as harvesting and storing grains at high moisture and reconstituted and ensiled grains, have been adopted [2] to maximize nutrient utilization, reduce production costs, and decrease environmental impacts. Reconstitution and ensiling processes allow for increased grain storage capacity during the harvest time and may reduce production costs and losses caused by fungi, insects, and rodents, which is very common in grains deposited in warehouses [10–12]. In addition, there may be an improvement in grain digestibility when grains are reconstituted and ensiled [13]. It is important to mention that storing grains in high moisture conditions may lead to similar or larger losses compared to storing dry grains if care is not taken to properly ensile the grains well (appropriate moisture content and compaction) and to cover it once ensiled.

We hypothesize that: (1) the ensiling time reduces insoluble N and increases NPN content in reconstituted corn and sorghum grains; and (2) diets based on reconstituted and ensiled grains will have greater microbial efficiency and greater intestinal and total digestibility compared to diets based on dry ground grains. Furthermore, we hypothesize that the processing method and grain source will not affect rumen pH. Thus, the objectives of the study were: (1) to evaluate the effect of ensiling time and grain source on the nitrogen fractions; and (2) to verify the influence of processing method and grain source on intake, microbial efficiency, rumen pH, and ruminal, intestinal, and total digestibility by young Nellore bulls.

## Material and methods

### Experiment 1

The experiment was conducted in the Experimental Feedlot of the Animal Science Department at the Universidade Federal de Viçosa, Viçosa, MG, Brazil.

#### Grain processing and experimental design

Flint corn with 74.51% vitreous endosperm [14] and sorghum grains used in Experiments 1 and 2 were acquired at the same time from the same source (Rações Ideal, Minas Gerais, Brazil), at an approximately 13% moisture content, and stored in metal bins until their use.

The processes described below were the same for each grain source (sorghum and corn). Approximately 50 kg grain were ground in a hammer mill (DMP-2, Nogueiras, São João da Boa Vista, São Paulo, Brazil) with a 3-mm sieve, homogenized manually, and an approximately 15 kg sample was obtained using a quartering technique. Ground corn and sorghum grain presented 0.90 mm of mean particle size. In addition, a subsample was collected and analyzed for dry matter (DM; method 934.01) [15]. Then, water was added until the DM decreased to approximately 65%. The grain was soaked, mixed in a cement mixer (Rental Mixer 400L, Menegotti Indústrias Metalúrgicas Ltda., Schroeder, Santa Catarina, Brazil) for approximately 5 min, and allocated into 30 subsamples of approximately 500 g each using a quartering technique. Three grain subsamples were randomly assigned 1 of 10 ensiling times: 0, 7, 14, 28, 45, 60, 90, 120, 180, or 360 days. All subsamples were ensiled in nylon-polyethylene bags (25 × 35 cm; Doug Care Equipment Inc., Springville, CA), and the air was evacuated from the bags using a vacuum sealer (Eco vacuum 1040, Orved, Italy). The bags were stored in the laboratory at room temperature (21–26°C). Therefore, the experiment consisted of 20 treatments (two grain sources × ensiling times) and 60 silos (three replications per treatment).

After the designated ensiling time was reached, bag silos were opened, and a subsample of approximately 250 g was collected from each bag using a quartering technique. Subsamples were immediately frozen at -80°C, lyophilized (Liobras, São Carlos, São Paulo, Brazil), ground in a knife mill (Tecnal, Piracicaba, São Paulo, Brazil) with a 1-mm sieve, and stored separately for further laboratory analyses.

The evaluation of the bag silos was carried out according to a completely randomized design in a 2 × 10 factorial scheme: two grain sources (corn or sorghum) and 10 ensiling times (0, 7, 14, 28, 45, 60, 90, 120, 180, or 360 days).

#### Chemical analyses

Samples from each bag silo were analyzed for DM and N, methods 934.01 and 981.10, respectively [15]. In addition, samples were analyzed in triplicate for NPN, soluble N, insoluble N, and neutral detergent insoluble nitrogen (NDIN), according to Licitra et al. [16]. Approximately 500 mg of the sample was treated with 50 mL of distilled water and allowed to stand for 30 min, followed by the addition of 10 mL 10% trichloroacetic acid and an additional 30 min incubation. The remaining residue was filtered through quantitative filter paper (100 g/m^2^), washed with water, and the residual N was determined [16]. The NPN was estimated by the difference between total N and residual N.

The insoluble N was determined after the treatment of 500 mg of the sample with borate-phosphate buffer (12.2 g NaH_2_PO_4_·H_2_O + 8.91 g Na_2_B_4_O_7_·10 H_2_O + 100 mL tert-butyl alcohol/L) for 3 h. The remaining residue was filtered through quantitative filter paper (100 g/m^2^), washed with water and the residual N was determined. The residual N represents the insoluble N. The difference between the total N and the residual N represents the total soluble N (NPN + soluble N). The soluble N was obtained by subtracting NPN from the total soluble N [16].

#### Statistical analysis

Data were analyzed using PROC MIXED of SAS version 9.4 (SAS Institute Inc., Cary, NC) following the general model:

$$Y_{ijk}=\mu +G_{i}+T_{j}+{\left(GT\right)}_{ij}+e_{ijk},$$

where Y_ijk_ is the observed measurement; μ is the overall mean; G_i_ is the fixed-effect of the i^th^ level of grain source; T_j_ is the fixed-effect of the j^th^ level of ensiling time; (GT)_ij_ is the fixed-effect of the interaction between the i^th^ level of grain source and the j^th^ level of ensiling time; and e_ijk_ is the random error associated with Y_ijk_, with e_ijk_ ~ N(0, σ_e_^2^). The fixed effects of grain source and ensiling time, as well as their interaction, were tested. Results were deemed significant when *P* ≤ 0.05.

### Experiment 2

This experiment was conducted in the Experimental Feedlot of the Animal Science Department at the Universidade Federal de Viçosa, Viçosa, MG, Brazil, following the recommendations of the Ethics Committee for Animal Use (protocol number 42/2016).

#### Grain processing

Approximately 90 days before the experiment began, approximately 3000 kg of each grain source was ground in a hammer mill (DMP-2, Nogueiras, São João da Boa Vista, São Paulo, Brazil) with a 3-mm sieve, and the DM content was measured (method 934.01) [15]. Then, water was added until the DM decreased to approximately 65%. The grain was soaked and mixed in a cement mixer (Rental Mixer 400L, Menegotti Indústrias Metalúrgicas Ltda., Schroeder, Santa Catarina, Brazil) for 5 min. The reconstituted grains were ensiled in round reinforced concrete pipe silos (1.0 m inside diameter × 1.0 m long × 8.0 cm wall thickness) with a mean density of 1000 kg of fresh material/m^3^. An additional 3000 kg of each grain was ground and stored dry. Therefore, two grains (corn and sorghum) with two processing methods (dry ground or reconstituted and ensiled) were used in this study.

#### Animals, facilities, and experimental design

Five rumen-cannulated young Nellore bulls (age = 8 ± 1.0 months; initial body weight [BW] = 262 ± 18.3 kg; final BW = 370 ± 20.2 kg) were used, distributed in a 5 × 5 Latin square design. Initially, the animals had been identified, treated for internal and external parasites, and housed in a Tie-stall barn with concrete floor and equipped with water and feed troughs. Bulls were tethered for the duration of the experiment. The experiment lasted 110 days, with five periods of 22 days. These periods were divided into two subperiods of 14 days for dietary adaptation [17] and 8 days for fecal, urinary, and omasal sample collection. Bulls were weighed on day 1 and 22 of each experimental period. The experiment was developed in a 2 × 2 + 1 factorial scheme.

#### Diets

Four diets were comprised of 28.4% corn silage, 10.7% supplement, and 60.9% dry ground corn, dry ground sorghum, reconstituted and ensiled ground corn, or reconstituted and ensiled ground sorghum. An additional diet comprised 45.0% corn silage, 10.7% supplement, and 44.3% dry ground corn was used (Roughage+). This additional diet is commonly used for medium and small feedlots in Brazil [6]. The proportions of feedstuffs and chemical composition of the diets are presented in Table 1. The diets were formulated according to BR-CORTE recommendations [18] to provide, approximately 134 g of CP/kg dietary DM and to support an average daily gain of 1.2 kg/day.

**Table 1:** Feedstuffs and chemical composition of experimental diets.

	Diets				
		Dry ground		Reconstituted and ensiled	
Item	Roughage+	Corn	Sorghum	Corn	Sorghum
Feed, % of dry matter					
Corn silage	45.0	28.4	28.4	28.4	28.4
Dry ground corn	44.3	60.9	-	-	-
Dry ground sorghum	-	-	60.9	-	-
Reconstituted and ensiled corn	-	-	-	60.9	-
Reconstituted and ensiled sorghum	-	-	-	-	60.9
Soybean meal	6.8	6.8	6.8	6.8	2.9
Mineral premix^1^	2.9	2.9	2.9	2.9	1.0
Urea + ammonium sulfate^2^	1.0	1.0	1.0	1.0	6.8
Dry matter, % as fed	43.4	54.1	53.9	46.5	46.8
Chemical composition % of dry matter					
Organic matter	92.9	93.0	93.1	93.0	92.9
Crude protein	12.7	13.4	13.4	13.4	13.3
Ether extract	4.0	4.2	3.1	4.3	3.0
Neutral detergent fiber^3^	28.2	20.8	20.8	19.2	19.2
Non-fiber carbohydrates^4^	50.6	56.3	57.5	57.8	59.0
Starch	44.5	51.8	52.1	51.9	51.5

^1^Premix guarantees (per kg of premix DM): 200–220 g of Ca, 10 mg of Co (Min), 500 mg of Cu (Min), 22 g of S (Min), 333 mg of Fe (Min), 178.41 mg of F (Max), 10 g of P (Min), 25 mg of I (Min), 17 g of Mg (Min), 1500 mg of Mn (Min), 1100 mg of monensin, 100 x 109 CFU of *Saccharomyces cerevisiae* (Min), 6.6 mg of Se (Min), 50 g of Na (Min), 100,000 IU of vitamin A (Min), 13,000 IU of vitamin D3 (Min), 150 IU of vitamin E (Min), and 2,000 mg of Zn (Min).

^2^Urea + ammonium sulfate in a 9:1 ratio.

^3^Neutral detergent fiber corrected for residual ash and residual nitrogenous compounds.

^4^Non-fiber carbohydrates = 100 − [(crude protein–crude protein from urea + urea) + neutral detergent fiber corrected for residual ash and residual nitrogenous compounds + ether extract + ash].

The corn and sorghum grains (both dry ground and reconstituted and ensiled), corn silage and the other ingredients were weighed separately, then mixed at the time of feeding. A total mixed ration was provided twice per day (08:00 and 16:00 h). Feed bunks were evaluated each day to quantify refusals and to adjust daily feed allowance to a maximum of 10% of refusals.

Diet ingredients were sampled daily during the collection period (from days 15 to 22) and stored at -20°C. In addition, during the collection period, samples of refusals were collected every morning immediately prior to first feeding. Feed bunks were totally cleaned, refusals were weighed, and a sample of approximately 250 g was obtained using a quartering technique. Refusal samples from all treatments were immediately frozen at -20°C. At the end of the experimental period, all samples were dried in a forced-air oven (55°C) for 72 h and ground in a knife mill with a 1-mm sieve. The daily samples of each diet ingredient were grouped equally in a subsample of 250 g for each period (air-dried basis, 55°C for 72 h). The daily refusal samples were grouped in a subsample of 250 g for each animal within each period. The proportion of daily refusal in the composite sample was based on the amount of each refusal verified daily divided by the total amount of refusals verified during the collection period (air-dried basis, 55°C for 72 h). Then, samples were packed in plastic bags for further laboratory analyses. The ingredient samples were analyzed individually and used to calculate dietary composition.

#### Total digestibility and microbial efficiency

The intake was recorded during the data collection period (from days 15 to 22). The daily intake of individual dietary components, such as intake of DM, OM, starch, etc., was corrected to refusal composition based on the following equation:

$$Intake\;(kg/day)=\frac{total\;offered\;(kg)-total\;refusal\;(kg)}{days\;of\;evaluation}$$

From days 15 to day 19 of the experimental period, 24-h fecal and urine output was determined for all bulls. Feces were collected from droppings on the concrete floor and placed in 30 L buckets. At the end of each collection day (24 h), the buckets containing the samples were weighed, homogenized, and a subsample was dried in a forced-air oven at 55°C for 72 h and then ground in a knife mill with a 1-mm sieve. Furthermore, a composite fecal sample from 5 days of collection was made for each animal per period, based on the DM content of the feces collected on the individual days, and then stored for further laboratory analyses.

Urine output was collected from days 15 to 19 (over a 24 h period) using collecting funnels, which were attached to animals and coupled with lead hoses to conduct urine to 20 L containers with 200 mL of 50% sulfuric acid. At the end of the first collection day, the total urine volume was measured using a 2 L measuring cylinder, placed in a 20 L bucket, and homogenized manually. Then, a subsample of 50 mL was collected using a 100 mL measuring cylinder and immediately frozen at -20°C. The previously added acid solution was excluded from all calculations, so the total urine volume and urine subsample comprised only urine. During the subsequent collection days, the same procedures for the collection and measurement of urine volume were adopted. However, the collected subsample was proportional to the total volume obtained on the first day (e.g., considering 10 L as the urine volume on the first day and a 50 mL subsample. Hence, if the volume of urine on the second day was 5 L, the subsample was 25 mL). After each collection day, urine subsamples were combined with the samples from the previous days and immediately frozen at -20°C. So, one urine sample was obtained for each animal within the period. Microbial efficiency was estimated as described by Barbosa et al. [19], in accordance with daily purine derivative excretion and measured by summation of urinary allantoin and uric acid excretion. Microbial efficiency was expressed as grams of microbial crude protein synthesized (gMCP) per kilogram of total digestible nutrients intake (kgTDN).

#### Partial digestibility

From days 17 to 22 of the experimental period, 5 g/day of Co-EDTA [20] was infused via ruminal cannula. The Co-EDTA was diluted in distilled water, and the infusion was performed with the aid of a peristaltic pump (BP-600.4; Milan Equipamentos Científicos, Colombo, Paraná, Brazil) at a rate of 115 mL/h. On the last 3 days of each period, eight punctual omasal digesta samples were collected, with a 9-h interval between collections. Omasal digesta samples were collected at 08:00 and 17:00 h (day 20); 02:00, 11:00 and 20:00 h (day 21); and 05:00, 14:00, and 23:00 h (day 22). Omasal digesta sample collection was performed as described by Huhtanen et al. [21] via ruminal cannula. At each collection time, 200 mL of omasal digesta simple was filtered (porosity of 100 μm, 44% of the surface, Sefar Nitex 100/44, Sefar, Thal, Switzerland). Solid and liquid phases were individually sampled and lyophilized (Liobras, São Carlos, São Paulo, Brazil). Then, the solid phase samples obtained from the eight punctual omasal collections were combined, weighted, ground in a knife mill with a 1-mm sieve, and stored for further laboratory analyses. The same process was adopted for samples from the liquid phase. It is worth to mentioning that laboratory analyses were done individually in the solid and liquid phases.

The reconstituted omasal digesta was considered the sum of both solid and liquid phases (DM basis). The composition of reconstituted omasal digesta was estimated considering the participation of the solid phase on the reconstituted omasal digesta multiplied by the composition of solid phase plus the participation of the liquid phase on the reconstituted omasal digesta multiplied by the composition of liquid phase (DM basis). DM and nutrient fluxes in the omasum were estimated using a double-marker system [22]. Therein, indigestible neutral detergent fiber (iNDF) was used as a solid-phase marker, and Co-EDTA was used as a liquid-phase marker [23]. Ruminal and intestinal digestibility were expressed in relation to the intake for each portion of the digestive tract.

#### Rumen pH evaluation

Rumen pH was evaluated for all bulls over a 120-h period (days 15 to 19 for each experimental period). The readings were performed every 15 min using an intra-ruminal bolus pH meter (Model: WellCow^TM^; Roslin, Scotland, UK). Rumen status was classified into three categories: acute acidosis (rumen pH below 5.2) [24, 25], subacute acidosis (rumen pH between 5.2 and 5.8) [26, 27], and normal (rumen pH above 5.8).

#### Chemical analyses

Samples of feedstuff, refusals, feces, and omasal digesta were analyzed for DM, ash, N, and ether extract (EE), according to AOAC method numbers 934.01, 930.05, and 981.10 [15] and AOAC method number 945.16 [28], respectively. The OM content was determined as the difference between DM content and ash content. CP content was calculated by multiplying the total N content by 6.25. The neutral detergent fiber was evaluated according to Mertens [29], without the addition of sodium sulfite and with the addition of thermostable α-amylase. The neutral detergent fiber content was corrected for residual ash and protein (apNDF). Estimations of NDIN followed recommendations of Licitra et al. [16]. The iNDF content was calculated after *in situ* incubation of the samples from 3 cannulated bulls using F57 bags (Ankom Technology, Macedon, NY) for 288 h [30]. Non-fiber carbohydrates were calculated according to Detmann and Valadares Filho [31]. The starch analyses were performed following the recommendations of Silva et al. [32].

In the urine samples, we determined the concentrations of uric acid (automatic biochemical analyzer; autoanalyzer, model BS200E, Mark Mindray; Model BS200E; Shenzhen Mindray Bio-Medical Electronics Co., Ltd., China) and allantoin, according to Chen and Gomes [33].

#### Statistical analysis

Data were analyzed using the PROC MIXED procedure of SAS version 9.4 following the general model:

$$Y_{ijkm}=\mu +{Diet}_{i}+a_{j}+p_{k}+e_{ijkm},$$

where, Y_ijkm_ is the observed measurement; μ is the overall mean; Diet_i_ is the fixed-effect of the i^th^ level of dietary treatment (5 levels); a_j_ is the random effect of the j^th^ animal (5 levels), with a_j_ ~ N(0, σ_a_^2^); p_k_ is the random effect of the j^th^ level of period (5 levels), with p_k_ ~ N(0, σ_p_^2^); and e_ijkm_ is the random error associated with Y_ijkm_, with e_ijkm_ ~ N(0, σ_e_^2^). A series of orthogonal contrasts were constructed in order to test biologically relevant questions for the fixed effect of Diet. The first contrast tested the difference between diet based on 55% concentrate and the average of the other four diets. Subsequently, the three remaining orthogonal contrasts were used to test the effects of grain source, processing method, and their interaction. Results were deemed significant when *P* ≤ 0.05 and trending when 0.05 < *P* ≤ 0.10.

## Results

### Experiment 1

**Fig 1:**
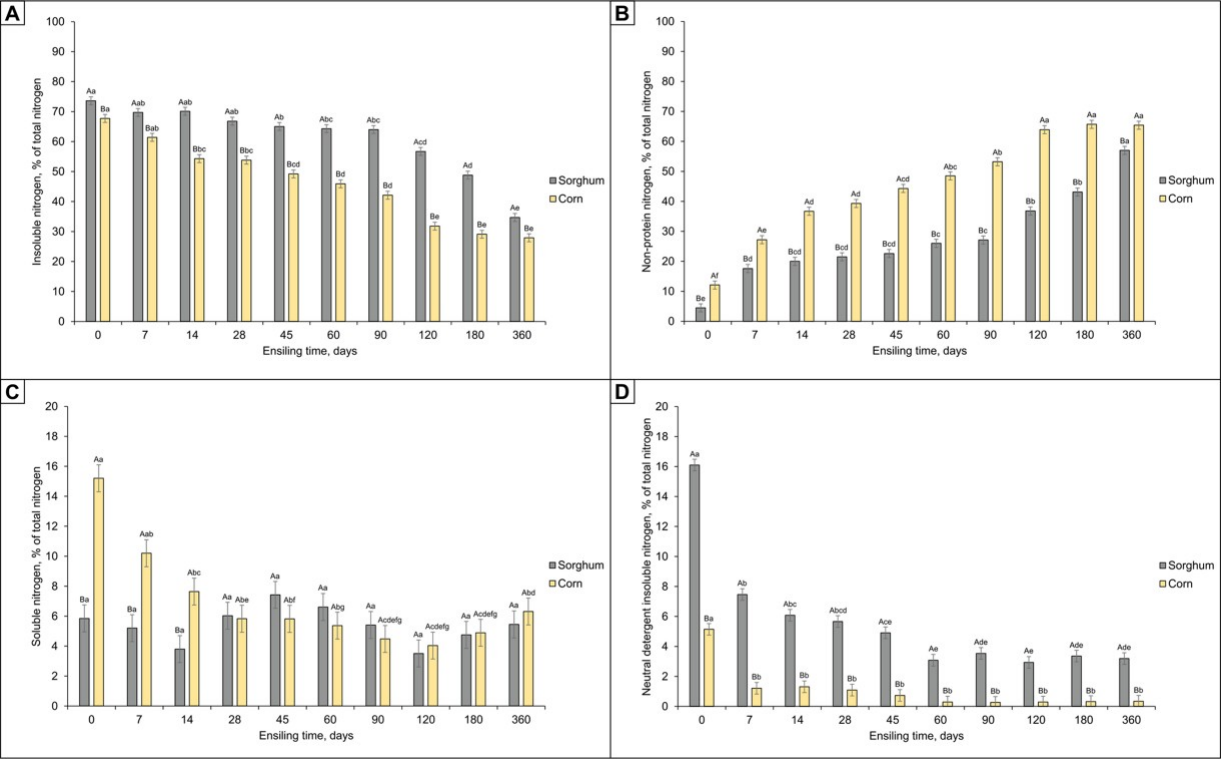
Effect of grain source and ensiling time on insoluble N (A), non-protein nitrogen (B), soluble N (C), and neutral detergent insoluble nitrogen (D) of reconstituted and ensiled grains. Grain source *P*-value, < 0.01; Ensiling time *P*-value, < 0.01; Interaction grain source and ensiling time *P*-value, < 0.01. ^A,B^ Upper case letters compare grains within ensiling time. ^a-g^ Lower case letters compare ensiling times within grain source. Means with different superscripts differ significantly at *P* ≤ 0.05.

There were interactions (*P* < 0.01) between the grain source and processing method for dietary N fractions (Fig 1).

The ensiling time did not affect (*P* > 0.10) the soluble N concentration for reconstituted and ensiled sorghum. On the other hand, the soluble N concentration decreased (*P* < 0.05) over time for reconstituted and ensiled corn. In addition, reconstituted and ensiled corn presented a greater (*P* < 0.05) soluble N fraction compared to reconstituted and ensiled sorghum from days 0 to 14 of ensiling. However, the soluble N concentration was the same (*P* > 0.10) in both corn and sorghum grains after day 28 of ensiling.

In general, there was a reduction (*P* < 0.05) in insoluble N concentration and an increase (*P* < 0.05) in the NPN fraction for both corn and sorghum during the ensiling time. However, reconstituted and ensiled corn showed lower (*P* < 0.05) insoluble N and greater (*P* < 0.05) NPN concentration compared to reconstituted and ensiled sorghum for all evaluated ensiling times.

There was a stabilization (*P* > 0.10) of the NDIN concentration after day 7 of ensiling for reconstituted and ensiled corn. For reconstituted and ensiled sorghum, there was a stabilization (*P* > 0.10) of the NDIN concentration after 45 days of ensiling. In addition, reconstituted and ensiled corn showed a lower (*P* < 0.05) NDIN concentration compared to reconstituted and ensiled sorghum for all evaluated ensiling times.

### Experiment 2

#### DM, OM, and apNDF

Intake as well as ruminal, intestinal, and total digestibility of DM, OM, and apNDF are presented in Table 2. The DM and OM intake in g/kg BW was not affected (*P* ≥ 0.18) by the evaluated factors. However, bulls fed diets based on reconstituted and ensiled grains showed lower (trend; *P* = 0.07; *P* = 0.06) DM and OM intake in kg/day compared to those animals fed diets based on dry ground grains. Grain source did not affect (*P* ≥ 0.77) apNDF intake in g/day and g/kg BW. On the other hand, animals fed dry ground grains presented grater (*P* ≤ 0.03) apNDF intake in kg/day and g/kg BW compared with those fed diets based on reconstituted and ensiled grains. Furthermore, bulls fed a diet based on 55% concentrate presented greater (*P* ≤ 0.01) apNDF intake in kg/day and g/kg BW compared with bulls fed remaining diets.

**Table 2:** Effect of concentrate level, grain source and processing method on Dry Matter (DM), Organic Matter (OM) and Neutral Detergent Fiber (apNDF) intake and digestibility of Nellore young bulls.

	Diet^1^									
		Dry ground		Reconstituted and ensiled			*P*–value			
Item	Roughage+	Corn	Sorghum	Corn	Sorghum	SEM	C	G	P	G × P
DM intake										
kg/day	5.75	6.06	5.74	5.00	5.62	0.663	0.47	0.63	0.07	0.14
g/kg of BW	18.90	19.45	18.72	16.77	18.71	1.473	0.67	0.56	0.21	0.21
DM digestion, %										
Rumen	43.00	50.45	52.58	43.56	48.63	2.691	0.04	0.15	0.04	0.54
Intestines	27.53	24.65	14.85	35.17	24.19	2.390	0.27	<0.01	<0.01	0.74
Total	70.53	75.20	67.43	78.73	72.82	1.671	<0.01	<0.01	<0.01	0.38
OM intake										
kg/day	5.33	5.69	5.38	4.68	5.25	0.620	0.80	0.63	0.06	0.13
g/kg of BW	17.54	18.26	17.57	15.69	17.50	1.379	0.79	0.56	0.18	0.21
OM digestion, %										
Rumen	49.85	56.50	59.10	50.45	55.44	2.187	0.03	0.08	0.03	0.56
Intestines	22.94	20.23	10.00	30.35	18.88	2.035	0.22	<0.01	<0.01	0.68
Total	72.79	76.73	69.10	80.80	74.32	1.682	0.01	<0.01	<0.01	0.55
apNDF intake										
kg/day	1.65	1.36	1.27	1.06	1.18	0.141	0.01	0.84	0.02	0.19
g/kg of BW	5.40	4.38	4.17	3.58	3.93	0.262	<0.01	0.77	0.03	0.22
apNDF digestion, %										
Rumen	44.21	50.21	49.41	41.27	45.47	2.476	0.38	0.48	0.01	0.31
Intestines	12.22	9.47	8.53	10.34	11.79	1.270	0.13	0.84	0.12	0.35
Total	56.43	59.68^a^	57.94^a^	51.61^b^	57.26^a^	2.244	0.93	0.33	0.04	0.07

C, effect of concentrate level (Roughage+ diet versus remaining diets); G, effect of grain source; P, effect of processing method; G × P, effect of interaction between grain source and processing method.

^1^Four diets were composed of approximately 28% corn silage and 72% concentrate (DM basis). An additional diet (Roughage+) was composed of 45% corn silage and 55% concentrate (DM basis).

^a,b^Within row, means without a common superscript significantly differ (*P* ≤ 0.05).

Ruminal DM digestibility was not affected (*P* = 0.15) by grain source. On the other hand, bulls fed diets based on sorghum showed greater (trend; *P* = 0.08) ruminal OM digestibility compared with those fed corn-based diets. Animals fed diets based on reconstituted and ensiled grains presented greater (*P* < 0.05) DM and OM ruminal digestibility compared with those fed diets based on dry ground grains. In addition, bulls fed a diet based on 55% concentrate presented lower (*P* < 0.05) ruminal DM and OM digestibility compared with those fed diets based on 72% concentrate. Grain source and concentrate level did not affect (*P* ≥ 0.38) ruminal apNDF digestibility. However, bulls fed diets based on reconstituted and ensiled grains presented lower (*P* = 0.01) ruminal apNDF digestibility compared with those fed diets based on dry ground grains.

The intestinal digestibility of DM and OM was lower (*P* < 0.01) for bulls fed diets based on sorghum compared with those fed diets based on corn. Similarly, bulls fed diets based on reconstituted and ensiled grains showed lower (*P* < 0.01) intestinal DM and OM digestibility compared with those fed diets based on dry ground grains. The concentrate level did not affect (*P* ≥ 0.22) intestinal DM and OM digestibility. In addition, the intestinal apNDF digestibility was not affected by any factor (*P* ≥ 0.12).

Total DM and OM digestibility was greater (*P* < 0.01) for bulls fed diets based on corn compared with those fed diets based on sorghum. Similarly, bulls fed diets based on reconstituted and ensiled grains showed greater (*P* < 0.01) total DM and OM digestibility compared with those fed diets based on dry ground grains. Furthermore, animals fed a diet based on 55% concentrate showed lower total DM and OM digestibility (*P* ≤ 0.01) compared with those fed the remaining diets. The concentrate level did not affect (*P* = 0.93) the total apNDF digestibility. However, there was a tendency (*P* = 0.07) for an interaction with regard to total apNDF digestibility. Bulls fed diets based on reconstituted and ensiled corn presented lower (*P* < 0.05) total apNDF digestibility compared with those fed a dry ground corn diet, but bulls fed diets based on reconstituted and ensiled sorghum presented similar (*P* > 0.10) total apNDF digestibility compared with those fed a diet based on dry ground sorghum.

#### CP and starch

The ruminal, intestinal, and total tract digestibility of starch and CP are shown in Table 3. The CP intake in kg/day and g/kg BW were not affected (*P* ≥ 0.12) by the evaluated factors. Bulls fed a diet based on 55% concentrate diet showed lower (*P* < 0.01) starch intake in kg/day and g/kg BW compared with those fed diets based on 72% concentrate. Similarly, bulls fed diets based on reconstituted and ensiled grains presented lower (*P* ≤ 0.04) starch intake in kg/day and g/kg BW compared with those fed diets based on dry ground grains. However, the grain source did not affect (*P* ≥ 0.45) starch intake.

**Table 3:** Effect of contrate level, grain source and processing method on Crude Protein (CP) and starch intake and digestibility, Total Digestible Nutrient (TDN) intake and microbial efficiency of Nellore young bulls.

	Diet^1^									
		Dry ground		Reconstituted and ensiled			*P*–value			
Item	Roughage+	Corn	Sorghum	Corn	Sorghum	SEM	C	G	P	G × P
CP intake										
kg/day	0.74	0.81	0.80	0.68	0.79	0.080	0.27	0.21	0.12	0.17
g/kg of BW	2.44	2.60	2.63	2.29	2.64	0.189	0.55	0.20	0.29	0.27
CP digestion, %										
Rumen	16.87	18.36	28.90	1.78	16.28	3.423	0.75	<0.01	<0.01	0.56
Intestines	54.75	57.11	42.69	77.82	58.23	3.848	0.60	<0.01	<0.01	0.42
Total	71.62	75.47	71.59	79.60	74.51	1.481	<0.01	<0.01	<0.01	0.46
Starch intake										
kg/day	2.30	3.04	2.91	2.52	2.77	0.299	<0.01	0.68	0.02	0.17
g/kg of BW	7.57	9.83	9.59	8.38	9.23	0.637	<0.01	0.45	0.04	0.19
Starch digestion, %										
Rumen	76.22	76.16	73.22	75.75	76.51	2.553	0.78	0.67	0.58	0.48
Intestines	15.58	12.27	4.29	22.36	10.92	1.675	0.12	<0.01	<0.01	0.32
Total	91.80	88.43	77.51	98.11	87.43	1.328	0.02	<0.01	<0.01	0.92
TDN intake	4.17	4.44	3.92	4.12	4.07	0.490	0.37	0.18	0.70	0.28
gMCP^2^/day	488	523	446	483	480	59.1	0.72	0.16	0.84	0.20
gMCP/kgTDN	117	118	114	117	118	3.8	0.77	0.29	0.28	0.11
gMCP/kgCPintake	658	645	557	710	608	20.4	0.12	<0.01	<0.01	0.69

C, effect of concentrate level (Roughage+ diet versus remaining diets); G, effect of grain source; P, effect of processing method; G × P, effect of interaction between grain source and processing method.

^1^Four diets were composed of approximately 28% corn silage and 72% concentrate (DM basis). An additional diet (Roughage+) was composed of 45% corn silage and 55% concentrate (DM basis).

^2^Microbial crude protein.

Animals fed diets based on corn presented lower (*P* < 0.01) ruminal CP digestibility compared with those fed diets based on sorghum. In addition, bulls fed diets based on reconstituted and ensiled grains presented lower (*P* < 0.01) ruminal CP digestibility compared with those fed diets based on dry ground grains. On the other hand, the concentrate level did not affect (*P* = 0.75) ruminal CP digestibility. Ruminal starch digestibility was not affected (*P* ≥ 0.58) by the evaluated factors.

Bulls fed diets based on corn presented greater (*P* < 0.01) intestinal and total CP and starch digestibility compared with bulls fed sorghum-based diets. Similarly, bulls fed diets based on reconstituted and ensiled grains presented greater (*P* < 0.01) intestinal and total CP and starch digestibility compared with those fed dry ground grains. On the other hand, the concentrate level did not affect (*P* ≥ 0.12) intestinal CP and starch digestibility. However, bulls fed a diet based on 55% concentrate showed lower (*P* < 0.01) total CP digestibility and greater (*P* = 0.02) total starch digestibility compared with those fed a diet based on 72% concentrate.

#### TDN and microbial CP

The TDN intake, microbial CP production (gMCP/day), and microbial efficiency were similar (*P* ≥ 0.16) for all evaluated diets. However, bulls fed diets based on corn had a gMCP/kgCP intake ratio that was approximately 16.3% greater (*P* < 0.01) compared with those fed diets based on sorghum. Likewise, bulls fed diets based on reconstituted and ensiled grains had gMCP/kgCP intake ratio that was approximately 9.7% greater (*P* < 0.01) compared with those fed diets based on dry ground grains. On the other hand, the concentrate level did not affect (*P =* 0.12) the gMCP/kgCP intake ratio.

#### Rumen pH

Rumen pH values below 5.2 were not observed for any diet. The average rumen pH, average rumen temperature, and the area above pH 5.8 were not affected (*P* ≥ 0.11) by the evaluated factors (Table 4). A grain source by processing method interaction (*P* = 0.02) was observed for the area between pH 5.8 and 5.2. Similarly, there were grain source by processing method interactions (*P* < 0.01) for the duration in min/day for pH above 5.8 and between 5.8 and 5.2. For bulls fed corn grain-based diets, the area under the pH curve between 5.2 and 5.8 was greater (*P* < 0.05) when grain was reconstituted and ensiled. This effect was not observed (*P* > 0.05) for bulls fed sorghum-based diets. Furthermore, bulls fed diets based on corn presented a lower (*P* < 0.05) time in min/day for pH above 5.8 and a greater (*P* < 0.05) time in min/day for pH between 5.8 and 5.2 when grain was reconstituted and ensiled. On the other hand, animals fed diets based on sorghum presented a greater (*P* < 0.05) time in min/day for pH above 5.8 and lower (*P* < 0.05) time in min/day for pH between 5.8 and 5.2 when grain was reconstituted and ensiled. The concentrate level did not affect (*P* > 0.36) any evaluated pH parameters.

**Table 4:** Effect of contrate level, grain source and processing method on pH and temperature of the rumen of Nellore young bulls.

	Diet^1^									
		Dry ground		Reconstituted and ensiled			*P*–value			
Item	Roughage+	Corn	Sorghum	Corn	Sorghum	SEM	C	G	P	G × P
Rumen pH	6.3	6.2	6.2	5.7	6.2	0.16	0.49	0.15	0.17	0.15
Rumen temperature	39.7	39.6	39.9	39.7	39.7	0.11	0.36	0.11	0.99	0.14
Area pH, Δ pH x H										
pH > 5.8	11.4	8.6	8.9	2.8	8.2	4.18	0.47	0.30	0.25	0.36
5.8 > pH > 5.2	0.3	0.7^b^	1.4^b^	5.4^a^	0.2^b^	1.11	0.81	0.08	0.21	0.02
pH ≤ 5.2	0.0	0.0	0.0	0.0	0.0	-	-	-	-	-
Duration pH, min/day										
pH > 5.8	1248	1184^a^^b^	980^b^	557^c^	1385^a^	139.8	0.71	0.03	0.38	<0.01
5.8 > pH > 5.2	192	256^b^^c^	460^b^	883^a^	55^c^	123.9	0.71	0.03	0.44	<0.01
pH ≤ 5.2	0.0	0.0	0.0	0.0	0.0	-	-	-	-	-

C, effect of concentrate level (Roughage+ diet versus remaining diets); G, effect of grain source; P, effect of processing method; G × P, effect of interaction between grain source and processing method.

^1^Four diets were composed of approximately 28% corn silage and 72% concentrate (DM basis). An additional diet (Roughage+) was composed of 45% corn silage and 55% concentrate (DM basis).

^a-c^Within row, means without a common superscript significantly differ (*P* ≤ 0.05).

## Discussion

### Experiment 1

The starch-protein matrix in cereal grains may limit starch digestion in ruminants [34]. However, during the ensiling process of reconstituted and ensiled grains, there is proteolysis of the starch-protein matrix [35]. The modification of dietary N fractions, such as reduction of insoluble N, may indicate proteolysis of the starch-protein matrix [35]. According to Junges et al. [35], bacteria account for 60.4% of the proteolysis in reconstituted and ensiled corn grains, followed by kernel enzymes, which contribute about 29.5%. In addition, fungi and fermentation products contribute to 5.3 and 4.8% of the proteolysis of protein bodies, respectively.

In the present study, corn and sorghum grains were submitted to the same physical processing and received similar amounts of water to reach 65% DM. Nevertheless, there were differences in the N fractions for the two evaluated reconstituted and ensiled grains. After day 14 of ensiling, there was a significant reduction in the levels of insoluble N (67.7 versus 54.3%) for corn grains, stabilizing after day 120 of ensiling (31.8%). Regarding sorghum, a minimum of 45 days of ensiling was necessary to obtain a significant reduction in the insoluble N fraction levels (73.6 versus 65.0%), and stabilization was not observed until day 360 of ensiling (34.7%). These findings indicate that the starch-protein matrix in sorghum grain may be more resistant to degradation compared with corn grain and/or other factors may be modifying the effectiveness of the proteolysis. Notably, the use of additives that aim to reduce the ensiling time of reconstituted and ensiled grains (i.e., proteolytic enzymes) may be advantageous, especially for sorghum grains. However, further research is needed to investigate how the dietary N fractions modifications can or can not affect starch availability or the quality of reconstituted and ensiled grains.

### Experiment 2

Although bulls fed reconstituted and ensiled grains showed numerically lower values, the DM, OM, and CP intake in g/kg BW were similar compared with those fed dry ground grains. However, bulls fed reconstituted and ensiled grain presented starch and apNDF intake in g/kg BW that were 9.3% and 12.2% lower, respectively, compared with bulls fed dry ground grains. Silva et al. [36], in a performance study that evaluated the same diets presented in this study, verified that bulls fed diets based on reconstituted and ensiled grains had a 12.8% lower DM intake compared with those fed diets based on dry ground grains. Furthermore, Caetano et al. [37] observed that increases in ensiled corn with a high moisture from 30 up to 45% of the DM in the diet resulted in quadratic declines in DM intake and linear reductions in metabolizable energy intake.

We observed no differences in the DM and OM intakes for the different concentrate levels, but the OM intake composition was different. Bulls fed a diet based on 55% concentrate presented reduced starch intake and increased apNDF intake compared with those fed diet based on 72% concentrate. Granja-Salcedo et al. [38] reported similar results: Bulk fill was not a limiting factor when the concentrate was increased from 30 up to 80%.

The increase in rapidly fermentable carbohydrates may explain the increase in the ruminal DM and OM digestibility of diets based on higher concentrate level. The ruminal OM and CP digestibility for bulls fed reconstituted and ensiled grains were 8.5 and 61.8% lower, respectively, compared with bulls fed diets based on dry ground grains. On the other hand, the intestinal digestibility of OM, CP, and starch were 62.7, 36.3, and 100.0% greater, respectively, for bulls fed diets based on reconstituted and ensiled grains compared with those fed dry ground grain diets. These results suggest that there is a shift in the digestion site from the rumen to the intestine for the aforementioned components for diets based on reconstituted and ensiled grains compared with those based on dry ground grains. In addition, the grain source may affect the site of digestion as well. The greater intestinal DM, OM, CP, and starch digestibility for bulls fed diets based on corn grain (reconstituted and ensiled or dry), may indicate a shift in the site of digestion for those diets compared with diets based on sorghum grain (either reconstituted and ensiled or dry).

A shift in the nutrient digestion site is considered an important factor to determine the form and absorption rate of the metabolites [39] and may influence the animals’ performance. Owens et al. [40] and Harmon and McLeod [41] reported higher energy efficiency when more starch is digested in the intestine compared with the rumen. Hence, animals that receive diets based on reconstituted and ensiled grain diets (corn or sorghum) in the current study would be more efficient than animals that receive dry ground grain diets (either corn or sorghum). These observations may be extended to animals fed diets based on corn (reconstituted and ensiled or dry) when compared with those fed diets based on sorghum diets (reconstituted and ensiled or dry).

The lower ruminal digestibility of starch for bulls fed diets based on reconstituted and ensiled grains was unexpected given that several studies [37, 42, 43] have been suggesting the opposite outcome. The higher ruminal digestibility of diets based on ensiled grains with high moisture verified by those studies is supported by the greater starch availability compared to diets based on dry grains [44–46]. However, other important factors that may affect the ruminal digestibility of grains must be considered [42]. Functional specific gravity, which is associated with gases and fluids present in the inter- and intracellular spaces of the feeds, influences the dynamics of the feed particles in ruminants [47]. Although commonly associated with particle size, feed hydration might also influence functional specific gravity. The reconstitution process in reconstituted and ensiled grains causes the spaces previously filled with gases in the grains to be filled by water, a phenomenon that increases the functional specific gravity and reduces the retention time of the digesta in the reticulum-rumen [48]. Besides that, proteolysis of the starch-protein matrix can increase the water uptake capacity of reconstituted and ensiled grain compared to the ground grain in its natural form [9, 44]. Although the starch is more amenable to ruminal digestion, perhaps the passage rate of starch in bulls fed a diet based on reconstituted and ensiled grain also increased compared with those fed diets based on dry ground grains. In that case, there would be no differences in ruminal starch digestibility and an increase intestinal digestion.

The increase in concentrate level from 55 to 72% did not affect the rumen pH and ruminal fiber digestibility. However, bulls fed a diet based on reconstituted and ensiled corn spent more than 14 h each day with a rumen pH below 5.8. The lower pH for a longer time may indicate subacute acidosis [26, 27]. Possibly the lower rumen pH for bulls fed reconstituted and ensiled corn was responsible for the reduction of the apNDF ruminal digestibility [26, 27]. In addition, the intake of apNDF in kg/day and g/kg BW was 14.8 and 12.2%, respectively, lower for bulls fed diets based on reconstituted and ensiled grains compared with those fed diets based on dry ground. According to Caetano et al. [45], the optimum forage NDF for maximum DM intake for diets based on ensiled grain with high moisture is around 21% higher compared with diets based on dry ground grains.

The lower ruminal and greater intestinal digestion of CP for bulls fed diets based on reconstituted and ensiled grains compared with those fed dry ground grains, is related to the greater efficiency of the use of this component [9, 13]. The reduction in ruminal CP disappearance observed for bulls fed diets based reconstituted and ensiled grains was offset by the increase in the gMCP:kgCP intake ratio; these data corroborate the greater efficiency of CP use. Differences in the nitrogen fractions of the grains caused by the reconstitution and ensiling process, may be responsible for the observed results.

Bulls fed a diet based on 55% concentrate showed a reduction in the total DM, OM and CP digestibility compared with those fed a diet based on 72% concentrate. However, the concentrate level did not affect apNDF total digestibility. Other studies [38, 49] have reported similar results. The improvement in diet quality when replacing forage by concentrate may explain these results.

The increase in total DM, OM, CP, and starch digestibility for diets based on ensiled grains with high moisture has been reported in the literature; this phenomenon is associated with an increase in animal feed efficiency [36, 45, 50]. Silva et al. [36], evaluating the same diets presented in this study, verified that bulls fed diets based on reconstituted and ensiled grains had a similar average daily gain (1.20 versus 1.22 kg/day) and greater (0.180 versus 0.158) feed efficiency (G:F) compared to those fed dry ground grains. Silva et al. [36] also verified that bulls fed diets based on corn grain had a similar average daily gain (1.28 versus 1.14 kg/day) and greater (0.181 versus 0.157) G:F compared to those fed sorghumgrain. Caetano et al. [37] verified that increases in ensiled corn with high moisture linearly improve carcass G:F. Furthermore, according to Caetano et al. [45], cattle fed ensiled corn with high moisture had a 13.9% greater G:F compared with those fed dry ground grains. In the present study, total digestibility of DM, OM, CP, and starch, expressed in kg/day (S1 Table), were not affected by the processing method. Consequently, even with the reduction in the intake, the total digested nutrients were not affected.

## Conclusion

The reconstitution and ensiling process reduces the insoluble N fraction of corn and sorghum grains and increases gMCP/kgCP intake ratio, intestinal, and total digestion of DM, OM, CP, and starch compared with dry ground process. Bulls fed diets based on corn grain show greater intestinal and total starch digestion compared to those fed sorghum grain. In addition, the increase in concentrate level from 55 to 72% does not affect the rumen pH, but it provides greater total DM, OM, and CP digestibility.

## Supporting information

S1 Table Effect of contrate level, grain source and processing method on total digestibility of dry matter, organic matter, crude protein, and starch. (DOCX)
