# Supplementary material for: Reconstituted and ensiled corn or sorghum grain: Impacts on dietary nitrogen fractions, intake, and digestion sites in young Nellore bulls
Source: PLoS One. 2020 Aug 7;15(8):e0237381. doi: 10.1371/journal.pone.0237381 (PMC7413414; doi:10.1371/journal.pone.0237381)
Supplement: S1 Table — (DOCX) [file pone.0237381.s001.docx]

**S1 Table. Effect of contrate level, grain source and processing method on total digestibility of dry matter, organic matter, crude protein, and starch.**

|  | **Diet^1^** | | | | |  |  | | | |
| --- | --- | --- | --- | --- | --- | --- | --- | --- | --- | --- |
|  |  | **Dry ground** | | **Reconstituted and ensiled** | |  | ***P* – value** | | | |
| **Item** | **Roughage+** | **Corn** | **Sorghum** | **Corn** | **Sorghum** | **SEM** | **C** | **G** | **P** | **G × P** |
| Total digestibility, kg/day |  |  |  |  |  |  |  |  |  |  |
| Dry matter | 4.06 | 4.54 | 3.91 | 3.97 | 4.08 | 0.497 | 0.89 | 0.58 | 0.65 | 0.41 |
| Organic matter | 3.88 | 4.35 | 3.75 | 3.80 | 3.90 | 0.471 | 0.89 | 0.56 | 0.63 | 0.42 |
| Crude protein | 0.53 | 0.61 | 0.58 | 0.58 | 0.59 | 0.065 | 0.49 | 0.84 | 0.65 | 0.52 |
| Starch | 2.09 | 2.68 | 2.25 | 2.47 | 2.41 | 0.251 | 0.01 | 0.05 | 0.82 | 0.13 |

C, effect of concentrate level (Roughage+ diet versus remaining diets); G, effect of grain source; P, effect of processing method; G × P, effect of interaction between grain source and processing method.

^1^Four diets were composed of approximately 28% corn silage and 72% concentrate (Dry matter basis). An additional diet (Roughage+) was composed of 45% corn silage and 55% concentrate (Dry matter basis).
